# The Interplay of Nutrition, the Gut Microbiota and Immunity and Its Contribution to Human Disease

**DOI:** 10.3390/biomedicines13020329

**Published:** 2025-01-31

**Authors:** Samantha L. Dawson, Emma Todd, Alister C. Ward

**Affiliations:** 1 School of Medicine, Deakin University, Waurn Ponds, VIC 3216, Australia; samantha.dawson1@deakin.edu.au (S.L.D.); s222375273@deakin.edu.au (E.T.); 2 Institute for Mental and Physical Health and Clinical Translation (IMPACT), Deakin University, Waurn Ponds, VIC 3216, Australia

**Keywords:** allergy, autoimmune disease, gut, immunity, inflammation, mental health disorders, microbiota, nutrition

## Abstract

Nutrition, the gut microbiota and immunity are all important factors in the maintenance of health. However, there is a growing realization of the complex interplay between these elements coalescing in a nutrition–gut microbiota–immunity axis. This regulatory axis is critical for health with disruption being implicated in a broad range of diseases, including autoimmune disorders, allergies and mental health disorders. This new perspective continues to underpin a growing number of innovative therapeutic strategies targeting different elements of this axis to treat relevant diseases. This review describes the inter-relationships between nutrition, the gut microbiota and immunity. It then details several human diseases where disruption of the nutrition–gut microbiota–immunity axis has been identified and presents examples of how the various elements may be targeted therapeutically as alternate treatment strategies for these diseases.

## 1. Introduction

The link between nutrition and the maintenance of health has been understood for some time [1] and the critical role of the immune system has been long realized [2]. However, only more recently with the rise of large-scale sequencing technologies has the importance of the body’s commensal microbiome been recognized, particularly the so-called ‘gut microbiota’ [3]. Indeed, the gut microbiota is now seen as a central link of an interconnected nutrition–gut microbiota–immunity axis that strongly influences health and its perturbation in various disease states.

Here, we provide an overview of the elements of the nutrition–gut microbiota–immunity axis and the important interplay between them, including how they impact key aspects of normal biology. We next detail a broad range of human pathologies associated with perturbation of the nutrition–gut microbiota–immunity axis, before exploring relevant therapeutic options that target elements of this axis. Finally, we present on-going challenges for the field.

## 2. The Triumvirate of Health: Nutrition, Gut Microbiota and Immunity

Nutrition, gut microbiota and immunity are individually complex topics, and each term has variable usage, therefore we provide a summary of each as used in this review.

### 2.1. Nutrition

Nutrients are the chemical components required by the body to function properly and maintain health, with most taken up via the gastrointestinal tract. This is critically dependent on the diet and its characteristics (e.g., the food matrix, food processing and preparation), nutrition status is also influenced by systemic factors (e.g., age, ethnicity, genetics, infection, etc.) and an interaction between gastrointestinal factors including luminal function, mucosa, barrier integrity, and the gut microbiota [4].

### 2.2. The Gut Microbiota

The gastrointestinal (GI) or gut microbiota represents the collection of microorganisms in the stomach, small intestine and large intestine, including bacteria, viruses, fungi and protozoa [5]. The definition of a ‘healthy gut microbiome’ (referring to the microbiota, genes, and metabolites) has evolved over time and is still highly elusive due to the varied interpretations of ‘healthy’ and high overall complexity [6]. Put simply, a healthy gut microbiome supports GI health and barrier integrity, digestion and immune response [6].

This review refers to microbiota and largely focuses on the community of bacteria in the gut. Microbial composition varies throughout the GI tract due to selective pressures stemming from the environment (e.g., pH levels, nutrient availability, etc.), microbial interactions and bacteriophages [7]. Compared to other sections of the GI tract, the large intestine is most abundant, harboring the largest number of gut microbes [7]. Constituents of the gut microbiota, including structural components and their nucleic acids, impact the host [8]. The gut microbiota also generates a wide range of bioactive products through a variety of metabolic processes, many of which also strongly affect host biology [9]. These include short-chain fatty acids (SCFAs), secondary bile acids, and hydrogen sulfide gases [10,11,12].

### 2.3. Immunity

The immune system is critical for the maintenance of health, responding to microbes, tumors and other insults. The innate or ‘non-specific’ arm comprises specific cell populations, notably including macrophages, neutrophils, mast cells, eosinophils, natural killer (NK) and other innate lymphoid cells [13]. These cells provide an immediate response to microbes and tissue damage typically triggered by pattern recognition receptors, including pathogen-associated molecular patterns (PAMPs) and damage-associated molecular patterns (DAMPs) [14]. It also includes anatomical barriers, such as the intestinal mucosa, as well as the complement system and acute inflammation [13,15]. The adaptive or ‘specific’ arm instead consists of various T and B cell populations that are critical for responses to non-self antigens, including immune memory upon re-exposure [16].

## 3. The Nutrition–Gut Microbiota–Immune Axis and Its Wider Impacts

**Figure 1:**
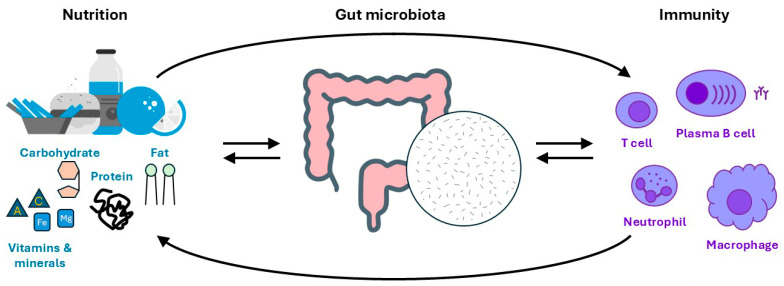
**The nutrition–gut microbiota–immunity axis.** Schematic representation of nutrition, the gut microbiota and immune cells, along with the reciprocal interactions between each of these components.

The relationships between nutrition, the gut microbiota and immunity have been determined in many ways, often identified in association studies involving patient cohorts, but causality often requires more contrived settings, with germ-free animal models in particular being critical in highlighting the central role of the microbiota [17], but with each component of the axis interconnected in a bidirectional manner (Figure 1). Thus, nutrition influences the gut microbiota, such as by selecting those able to utilize certain metabolites, while the gut microbiota influences immunity, often through structural components and metabolic products. However, nutrition can also impact immunity directly, with specific immune populations having unique requirements. Furthermore, immunity impacts the gut microbiota, and both can influence nutrition status through a variety of feedback mechanisms.

### 3.1. Nutrition–Gut Microbiota Interactions

Diet strongly influences the gut microbiota across the lifespan. Recent evidence indicates that the maternal diet during pregnancy impacts early infant gut microbiota assembly [18]. Following birth, infant feeding further modulates the gut microbiota and its functional capacity [19]. For example, the gut microbiota of breastfed infants is lower in diversity and enriched for genes that degrade breastmilk sugars [20] but is also primed to break down plant materials upon their introduction to the infant diet due to the unique polysaccharide diversity of human breastmilk [21]. The transition to solid foods further drives the maturation of gut microbiota: by 12 months of age, the infant gut microbiota increases in complexity with enrichment for genes that degrade complex sugars and starch [20]. By three years of age, the diversity of the child gut microbiota more closely resembles that of adults [22].

In adulthood, diet continues to act as a key selective pressure within the gut environment. Dietary protein and carbohydrate are key drivers of gut microbiota composition and function, with low dietary carbohydrates selecting for microbes able to utilize host carbohydrates like mucins, and low protein increasing competition for host-derived amino acids, drastically changing the shape of the gut microbiota community [23]. Gradients of carbohydrate availability along the large intestine select for unique microbial communities within these micro-environments [21]. Excess dietary protein may result in protein-derived molecules reaching the intestine where they raise the pH, selecting for microbes that can perform proteolytic rather than saccharolytic fermentation, and promoting the production of microbial metabolites such as ammonia, polyamine, and branched-chain fatty acids [24]. Dietary fat consumption can also exert selective pressure on the gut microbiota, through the increased production of anti-microbial bile acids to solubilize the fat, selecting for microbes that can tolerate and/or metabolize them [11,12]. Conversely, high fiber intake can mitigate this by sequestering bile acids and cholesterol, limiting opportunities for microbial activity [12].

The gut microbiota can conversely affect host nutrition through numerous mechanisms. Gut microbes can synthesize several key vitamins de novo, including B vitamins such as folate, riboflavin (B_2_) and B_12_, [25,26], as well as vitamin K [26]. Gut microbiota may also impact the absorption and bioavailability of vitamins and minerals either positively, such as through the production of phytases which release usable forms of calcium, magnesium, or phosphate, or negatively, through lipopolysaccharide-induced inhibition of vitamin C absorption [27]. There also appears to be a bi-directional relationship between gut microbiota and fat-soluble vitamins, with microbes modulating the production, absorption, transport, and activation of many of these nutrients, whilst their consumption through diet may influence microbiota composition and the microbiota-immune interface [28]. Fermentation of otherwise indigestible polysaccharides by the gut microbiota results in the production of SCFAs—principally acetate, propionate, and butyrate—that can account for ~10% of total daily energy requirements, forming the primary energy source for colonocytes [29,30]. Certain microbes have the capacity to increase the bioavailability of specific plant-derived compounds, such as polyphenols [31]. The relationship between polyphenols and gut microbiota is complex, with certain gut microbes sharing the metabolic processes for their uptake with human cells, whilst these compounds also form a selective pressure by acting either as a prebiotic (mostly for lactic acid bacteria) or as an antimicrobial [32].

Gut microbes also complement the acquisition of amino acids from the diet by increasing their bioavailability [33]. They complement human protein catabolism into amino acids and peptides and perform de novo synthesis of some amino acids which can be taken up into microbial cells or used by the host [34]. However, some microbial metabolites from proteolytic fermentation can negatively affect host amino acid transport and absorption [34]. The gut microbiota also contributes to the production of indole derivatives, polyamines, secondary bile acids, and polysaccharide A [35].

Lastly, the gut microbiota may influence host nutrition status and diet choices by modulating physiological responses to food. For example, microbial metabolites impact satiety, hyperphagic drive, fatty acid oxidation and gastric emptying through gut hormones such as leptin, ghrelin, and glucagon-like peptide 1 (GLP-1) as well as through neurotransmitter, neuropeptide and receptor gene modulation (e.g., GPR41/43, CCK, endocannabinoids) [36,37]. A systematic review identified several taxa and metabolites positively (*Coriobacteriaceae, Veilloellaceae, Prevotella, Coprococcus,* and *Ruminoccocus*), negatively (SCFA, *Streptococcus*, butyric acid, *Eubacterium rectale* group, *Bacteroidetes*: *Firmicutes* ratio, *Faecalibacterium*, *Prevotellaceae*, *Escherichia*, and *Shigella*), or inconsistently (*Bacteroides*, *Bifidobacterium*, *Lactobacillus*) associated with ghrelin [38]. Gut microbes may also influence food reward and satiety through molecular mimicry of endogenous peptides such as insulin, neuropeptide-Y, and melanocyte-stimulation hormone [37]. Clinical trials have also shown that gut microbiome composition and function also influence post-prandial responses to foods and thus their impact on host nutritional status [39,40].

### 3.2. Gut Microbiota–Immunity Interactions

The gut microbiota can impact immunity in several ways. The early life microbiota has been shown to be pivotal in the maturation of the immune system [41,42]. Most notably, it influences the production of regulator T (Treg) cells that control tolerance, and helper T (Th)17 cells that regulate immunoglobulin affinity and class switching, the generation and activity of various innate immune cell populations as well as intestinal barrier function [43].

The gut microbiota continues to influence the production and function of immune cell types throughout life [44]. This is principally mediated via microbiota-derived PAMPs acting via Toll-like receptors (TLRs) and nucleotide-binding oligomerization domain-containing proteins (NODs) [45], as well as microbiota-derived metabolites, especially the SCFAs butyrate and propionate [10], which collectively can act both locally and systemically.

With respect to immune cell production, ligands for NOD1 are sensed by bone marrow stromal cells leading to the expression of the cytokines, such as interleukin (IL)-6), IL-7, stem cell factor and thrombopoietin to promote the production of both innate and adaptive immune cells [46]. Ligands for TLR4 are sensed by intestinal epithelial cells stimulating expression of IL-17 that in turn induces granulocyte colony-stimulating factor (G-CSF) to stimulate neutrophil production [47,48], a pathway also stimulated by gut-segmented filamentous bacteria directly [49]. Meanwhile, ligands for TLR1/2 promote balanced Th cell generation largely via promoting the production of Treg cells [50]. Treg differentiation and homeostasis are also regulated by SCFAs [51,52] and bile metabolites [53], while SCFAs are also important for sustaining B cell metabolism that underpins antibody production [54].

Functionally, SCFAs play a myriad of roles, acting mainly to suppress inflammation [35]. In neutrophils, they serve to moderate effector functions [55] and pro-inflammatory cytokine expression [56]; in macrophages, they reduce pro-inflammatory mediator production [57] and instead elicit an antimicrobial transcriptional program [58]. They also inhibit mast cell activation [59] and limit eosinophil trafficking and survival [60]. However, they can also act in ways to enhance inflammation [61]. Other metabolites are also important, with bile metabolites shown to exert anti-inflammatory effects in monocytes, macrophages and NK-T cells [62], and polyphenols being both anti-inflammatory and anti-oxidant [31]. The gut microbiota can also affect epithelial barrier integrity via a direct effect of SCFAs on intestinal epithelial cell differentiation [10], or indirectly as a consequence of its effect on intestinal inflammation [63].

The gut microbiota–immunity interaction is bi-directional, with immunity influencing the gut microbiota by several mechanisms [64]. For example, B cells produce considerable quantities of immunoglobulin (Ig)A against commensal bacteria [65], while the intestinal mucosa produces mucin [13]. Together these molecules forming a protective barrier in the intestinal lumen neutrophils serve to modulate the microbiota, being recruited to the mucosal epithelium to constrain segmental filamentous bacteria (SFB) largely through inducing expression of antimicrobial proteins (AMPs) by the intestinal mucosa [66], and moving to the lumen to control microbial overgrowth mainly via reactive oxygen species (ROS) production [67,68]. Conversely, by-products of their action can promote the growth of specific species, such as tetrathionate in the case of *Salmonella* [69] and nitrate in the case of *Escherichia coli* [70]. A population of innate lymphoid cells (ILCs) called mucosal-associated innate T (MAIT) cells recognized metabolites of bacterial riboflavin biosynthetic pathways to elicit antibacterial responses that modulate microbiota composition [71]. Finally, Treg cells have been shown to be important in controlling the composition of the microbiota, with a key role in enforcing commensalism [72,73].

### 3.3. Nutrition–Immunity Interactions

Nutrition strongly influences the immune system in a number of ways, with the involvement of both micro- and macro-nutrients, particularly specific amino acids, through vitamins and trace elements. Thus, both vitamins A and D can impact the immune system in several ways. They separately support the induction of Treg cells and IL-22-producing ILCs at mucosal sites but suppress T cell expression of IL-17 and interferon (IFN)γ, while they have differential effects on homing of T cells to the gut [74]. They are also important for barrier function through the expression of tight junction proteins on intestinal epithelial cells [75]. Iron modulates the Ig class switch in B cells [76], while siderophores prime innate immune cells [77]. Micronutrients and protein are critical for T cell production and polarity [78], with malnutrition associated with Th2 skewing and inflammation [79]. Fatty acid composition can also influence immune function, with saturated and trans-fats shown to be pro-inflammatory, while monounsaturated fats and polyunsaturated omega-3 fats anti-inflammatory [80]. Finally, polyphenols and phenolic compounds can interact with the immune system to exert anti-inflammatory effects [81,82].

The immune system can also influence nutrition. This is particularly true of inflammation that causes a range of physiological changes sometimes referred to as ‘nutritional immunity’ that limit uptake of key micronutrients, notably including iron and vitamin A [83].

### 3.4. Impacts on Other Body Systems

The diet-gut microbiome-immune axis can also impact other body systems. For example, SCFAs can impact central nervous system (CNS) development and function [84,85], as can inflammation [86]. Serotonin, a neurotransmitter, and tryptophan levels are modulated via the diet-gut microbiome-immune pathway. The essential amino acid tryptophan can be used for the synthesis of serotonin by enterochromaffin cells (ECs) in the GI, but more than 90% is catabolized via the kynurenine pathway [87,88], resulting in neuroprotective kynurenic acid and neurotoxic quinolinic acid [87,88]. Some bacteria can promote ECs to synthesize serotonin, and some can synthesize it from tryptophan [87]. Moreover, gut microbiota metabolites and cell components modulate tryptophan levels, by activating or suppressing the kynurenine pathway; for example, SCFAs suppress tryptophan degradation via this pathway, whist lipopolysaccharide (LPS) can activate TLRs that initiate the kynurenine pathway [87]. An increased kynurenine to tryptophan ratio has been suggested as a proxy for an activated immune system [89]. However, manipulating the gut microbiota via probiotic supplementation can reduce the ratio of kynurenine to tryptophan [90].

Offspring of germ-free rodents exhibit many developmental disturbances, for example, in axonogenesis [91], microglial development [92], social behavior, blood–brain barrier permeability [93], brain signaling mechanisms [94] and allergy susceptibility [95]. These deficits may occur, in part, via an absence of microbial metabolites [91,96]. In some cases, inoculating germ-free mice with bacteria and/or SCFAs partially corrects some developmental defects, for example, blood–brain barrier permeability [93], microglial defects [92], and fear behavior [97]. There does however appear to be a critical window for re-establishment that occurs early in the postnatal period [97], likely due to an early window of high developmental plasticity [98].

## 4. Disruption of the Nutrition–Gut Microbiota–Immunity Axis and Its Impact

**Figure 2:**
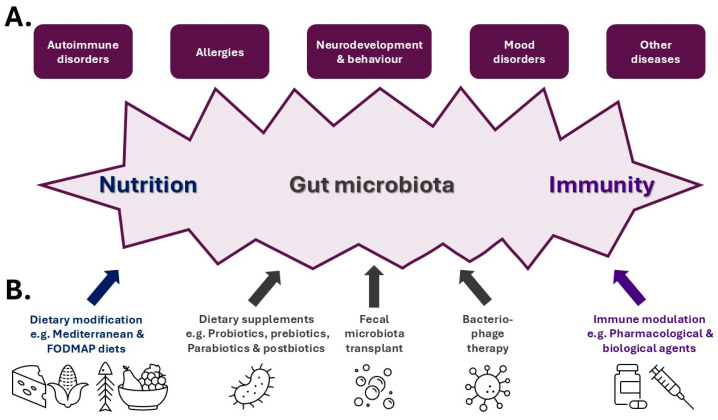
**The impacts of a disrupted nutrition–gut microbiota–immunity axis and its therapeutic targeting.** (**A**) Schematic representation of the impacts that result from disruption of the nutrition–gut microbiota–immunity axis with (**B**) examples of how each element of the axis may be modulated therapeutically.

There is considerable evidence that the nutrition–gut microbiota–immunity axis impacts humans in many ways, several of which we review here (Figure 2A). However, these impacts can also affect the gut microbiota and/or immunity and in some cases nutrition, which can make causality difficult to definitively determine [99]. Moreover, the majority of these impacts are heavily influenced by genetic background and environmental factors that often represent trigger(s) [99]. In addition, several of the impacts are interrelated, such as both autoimmunity and allergic diseases with mental health disorders [100,101], for example, dermatological autoimmune diseases and depression [102] as well as atopic dermatitis and attention-deficit/hyperactivity disorder (ADHD), especially its more severe forms [103]. This further suggests a common etiology.

Globally, diet quality is generally low and has only modestly improved over the past 30 years [104]. Poor diet quality is likely a significant disruptor of the nutrition–microbiome–immune axis. The Western dietary pattern (e.g., low in fiber, nutrient-dense foods and high in salt, sugar and saturated fat) increases colonic mucosal inflammation, reduces colonic butyrate and/or fiber fermenting butyrate producers, and promotes bile tolerant genera such as *Bilophila wadsworthia* (a hydrogen sulfide producer) [105,106,107]. There is also increasing concern surrounding the rise in inflammatory and auto-immune diseases [108,109], and circulating antibody levels [110] in the Western world. It is postulated that these changes may be driven by alternations in microbiota diversity and composition [111], with concern that aspects of modern life such as dietary composition, lack of environmental interaction, and antimicrobial exposure may play a role in these alterations. Evidence has been sought by comparing the microbiota of non-industrial traditional populations to those of Western individuals [112,113] and those of recently migrated people to naturalized citizens of Westernized countries [114]. Potential mechanisms for these alterations include diet-mediated microbial extinctions [115] and country-specific antimicrobial resistome profiles [116] creating selective pressures in the modern world.

### 4.1. Autoimmune Disorders

Autoimmune diseases are a consequence of an inappropriate adaptive immune response to a self-antigen leading to chronic inflammation and host tissue damage [117]. Common examples are inflammatory bowel diseases, such as Crohn’s disease (CD) and ulcerative colitis (UC), rheumatic diseases, such as rheumatoid arthritis (RA), systemic lupus erythematosus (SLE) and psoriatic arthritis, as well as celiac disease, multiple sclerosis, type 1 diabetes, vasculitis and alopecia areata [118]. The diet, gut microbiome and immune system have all been implicated in concert along with genetic [119] and environmental factors [120].

A role for immunity in autoimmune diseases is explicit, with a central role for autoreactive B and T cells and autoantibodies, but with activated T cells, neutrophils and macrophages often responsible for the pathology [118]. However, microorganisms represent key contributors via molecular mimicry leading to cross-reactive autoantibodies, through non-specific immune stimulation by PAMPs that potentiates a pre-existing adaptive response or through impacts of metabolites [121]. While specific microbes can be a sole trigger, such as Group A *Streptococcus* via molecular mimicry in rheumatic fever [122], more typically autoimmune diseases have been associated with broader microbial changes and impacts. In inflammatory bowel disease (IBD) and CD, disturbances in the microbiota have been identified, particularly an increase in facultative anaerobes (such as *Bacilli* and *Enterobacteriaeceae*) at the expense of obligate anaerobes (such as *Clostridia* and *Bacteroidia*) [99]. There are also alterations in mucosal T cell populations leading to excessive immune responses to the normal gut flora, leading to inflammation and associated with impaired barrier function, which collectively mediate pathological changes [99]. Disease activity in Sjorgen’s syndrome was found to be inversely correlated with microbiota diversity [123]. Disrupted gut microbiota has also been implicated in SLE [124], with the expansion of *Ruminococcus gnavus* associated with disease activity [125]. A similar expansion has been observed with *Prevotella copri* in RA [126], in this case with cross-reactive autoantibodies [127].

Nutrition can also contribute to autoimmune diseases through a range of mechanisms, with diet a key aspect [96]. This can be by supplying specific triggers, such as gluten in the case of celiac disease [128], but also substrates for microbiota metabolism that is associated with autoimmune disease such as tryptophan-derived kynurenine [129] or fiber-derived SCFAs [10].

### 4.2. Allergies

Allergies represent a diverse group of disorders, including atopic dermatitis, asthma, food allergy and psoriasis, which represent a significant health burden that is on the rise [130]. The signature feature of an allergic response is the induction of a pathogenic response to otherwise harmless exogenous antigens [131]. This involves a so-called type 2 immune response characterized by excessive inflammation [132]. There is a predominance of helper Th2 cells and IgE-producing B cells in concert with high levels of the cytokines IL-4, IL-5, IL-9 and IL-13 associated with recruitment and activation of mast cells, eosinophils and other leukocytes [133]. Alterations in Treg cells and other T cell subsets are particularly important [134]. Both nutrition and gut microbiota have been implicated in allergies. Malnutrition with respect to both macro- and micro-nutrients represents a common causative agent across all allergy types, which can be exacerbated by allergens acting as nutrient binders [135]. Gut microbiota disturbance has also been implicated in tuning the immune response toward allergic disease [136], as reported, for example, in atopic dermatitis [137]. Indeed, alterations in the gut microbiota in early life have been shown to strongly influence immune development within the gut mucosal tissues, the critical site for food allergy. In particular, the gut microbiota impacts the so-called RORγt+ subset of Treg cells that ultimately leads to the Th2 response [43]. However, it can also impact the epithelial barrier, causing increased permeability and so allergen translocation [43]. In addition, the metabolites produced by gut microbiota have been implicated in atopic dermatitis [138], asthma [139] and rhinosinusitis [Lee, 2018}, thought to be mediated via impacts on the immune system. Maternal gastrointestinal carriage of *Prevotella*, a genus of commensal bacteria that produces SCFA and the TLR4 ligand endotoxin was shown to negatively correlate with food allergy in offspring, with the association greatest for mothers that consumed high total intakes of dietary fat (>77 g/day) plus fiber (>22 g/day) [140].

### 4.3. Neurodevelopment and Social-Emotional Behavior

In addition to genetic, diet, lifestyle and environmental factors, the gut microbiota and their SCFA metabolites influence brain function and behavior via their interaction with the immune and endocrine systems, neuronal pathways, neurotransmitter pathways, including the hypothalamus–pituitary–adrenal (HPA) axis [141,142,143]. Many observational studies report relationships between the state of the infant gut microbiota and later behavioral [144,145], temperamental [146,147] and cognitive outcomes [148]; however, it is still unclear whether these differences are causes or consequences due to the bi-directional connection between the gut and brain.

Preclinical studies in rodents demonstrate that high-fat diets (typically 60% energy from fat) disturb the gut microbiota during gestation and impair brain development and behavior in offspring compared to standard chow [149,150]. Male offspring of dams exposed to a high-fat diet had cognitive and behavioral disturbances compared to controls [149]. Similarly, Buffington et al. [150] showed that a high-fat diet during gestation depleted *Lactobacillus reuteri* (a lactic acid bacteria considered to be probiotic) in dams and offspring, and disturbed brain development and social behavior. Importantly, these 60% high-fat gestational diets (60%) have been found to impact the gut-immune axis in offspring, by disturbing offspring gut microbiota, impairing offspring intestinal development and barrier function, and increasing inflammatory cytokine production [151]. Buffington et al. corrected the diet-induced disturbances in the offspring gut microbiota by administering *L. reuteri*, which in turn, ameliorated some of the deficits in oxytocin levels and social behavior [150]. Importantly, the specific types of fat were not typically detailed, therefore it is unclear whether all types of dietary fat would have this effect, particularly omega-3s, which have anti-inflammatory potential.

In pregnancy, diet quality has a small positive association with neurodevelopmental outcomes, particularly for child cognition [152] and ADHD risk [153]. One study investigated the maternal gut microbiota in the third trimester of pregnancy on these relationships, finding that mothers of children with elevated behavior problems had a lower alpha diversity and lower abundances of butyrate-producing genera in pregnancy compared to mothers of children with normative behavior [154]. Importantly, the diversity of the gut microbiota in pregnancy indirectly mediated the association between prenatal diet quality and child emotional behavior. Given that both maternal [154] and infant [145] gut microbiota relate to these behavioral outcomes, and that the maternal diet alters the infant gut microbiota [18], a new random controlled trial (RCT) study is now underway targeting prenatal diet-by-microbiome pathways to support child neuropsychiatric outcomes (ACTRN12623001343695).

In humans, it has long been understood that maternal immune activation (MIA) induced via influenza or infection disturbs brain development [155,156]. Elevated maternal pro-inflammatory cytokines such as Interleukin 6 and tumor necrosis factor alpha increase neuroinflammation in the fetal brain and activate microglial cells [155,156,157]. In animal models immune-mediated neurodevelopmental disorders, polyriboinosinic-polyribocytidylic acid (poly(I:C)) is used to induce MIA [158]. In mice, MIA disturbed the maternal and offspring gut microbiota resulting in increased colonic IL-6, intestinal permeability, and behavioral abnormalities in offspring [159]. Importantly, these disturbances were partially corrected in offspring by administering *Bacteroides fragilis* [159], a bacteria that produces a bacterial polysaccharide that corrects T cell deficiencies and Th1/Th2 imbalances [160]. In the reverse direction, some gut microbiota such as segmented filamentous bacteria or a set of human commensals that promote Th17 can catalyze MIA when present during gestation resulting in neurodevelopmental defects in offspring mice [161].

### 4.4. Mood Disorders

Mental disorders place in the top ten leading causes of disease burden globally, with mood disorders such as depressive disorders being the second leading contributor to years lived with disability (YLD) and anxiety disorders being the eighth [162]. In adults, there is evidence of overall compositional differences in the gut microbiota of those with and without major psychiatric disorders (such as major depressive disorder (MDD), bipolar disorder and schizophrenia) [163]. In particular, higher levels of bacteria that produce lactic acid, and bacteria involved in glutamate and GABA metabolism were observed, and lower levels of SCFA-producing bacteria in those with mental disorders compared to those without [163]. Elsewhere, compared to healthy controls, those with unmedicated MDD had increased levels of *Enterobacteriaceae*, a pro-inflammatory bacteria, and lower levels of SCFA-producing bacteria [164]. Importantly, *Enterobacteriaceae* was associated with abnormalities in the functional connectivity of the hippocampus in MDD, highlighting potential disturbances in inflammatory pathways. An early study showed that transferring gut microbiota from depressed patients and healthy controls are transferred to antibiotic-treated rats, those receiving microbiota from depressed donors exhibit depressed and anxiety-like behavior, elevated fecal acetate and an elevated kynurenine to tryptophan ratio, which is suggested as a marker of immune activation [89]. However, since then, this study and others have been criticized for methodological concerns, and it is anticipated that there is significant publication bias in the literature [165]. Poor-quality diets, including diets with high pro-inflammatory potential, have also been associated with greater depression risk in adults [166].

GI disturbances [167], immune dysfunction and elevated low-grade inflammation [168] are commonly observed in psychiatric disorders, with the latter two linked to disease progression in neuropsychiatric disorders [168]. Markers of inflammation, such as C-reactive protein (CRP), IL-12, are significantly elevated in patients with depression compared to those without depression, even after accounting for confounding factors [169]. Although there are distinct compositional differences between those with mental disorders and those without, to date the evidence supporting specific microbial targets for treatment or prevention is still in its infancy. There are associations between diet and mental health likely due to the anti-inflammatory effect of a healthy diet, potentially mediated via the gut microbiome [170], but this requires further confirmation.

## 5. Therapies Targeting the Nutrition–Gut Microbiota–Immunity Axis

With the growing appreciation of the nutrition–gut microbiota–immunity axis and its influence in maintaining the balance between health and disease, a range of therapeutic strategies are being developed that target a specific axis component to ameliorate disease (Figure 2B).

### 5.1. Dietary Modulation

Given the role of the gut microbiota in human metabolism, different dietary patterns shape the gut microbiota in distinct ways. We present examples from Mediterranean dietary interventions as this is considered a ‘gold standard’ diet for disease prevention [171], and the low fermentable oligosaccharides, disaccharides, monosaccharides and polyols (FODMAP) diet, which is a proven therapeutic diet for irritable bowel conditions.

The Mediterranean diet is a healthy dietary pattern that is associated with reduced risks for many non-communicable diseases having inflammatory origins [172]. The Mediterranean diet is fiber-rich and characterized by high intakes of polyphenol-containing vegetables, fruit, whole grains, olive oil, legumes and nuts and moderate to low intakes of dairy and red meat. It has long been established that the Mediterranean diet has an anti-inflammatory impact through reducing CRP, IL-6 and intracellular adhesion molecule-1 leading to improved endothelial function [173]. Some studies have reported changes in the gut microbiota that correspond with a less inflammatory state, for example, compared to controls following their habitual diet, individuals on the Mediterranean diet showed reduced *B. wadsworthia*, a pro-inflammatory hydrogen sulfide-producing bacteria, and increased genes for butyrate metabolism [174], and the abundance of SCFA-producing bacteria such as fiber-degrading *Faecalibacterium prausnitzii* [174], *Roseburia* spp. [174], *E. rectale* [175], *P. copri*, amongst others [175]. Additionally, a 12 month Mediterranean dietary intervention reduced inflammatory markers (CRP and IL-17) and reduced the production of secondary bile acids; this study noted an increase in the abundance of SCFA-producing bacteria [175]. In another study, a 3-month Mediterranean dietary intervention improved intestinal barrier integrity in women with barrier impairment, in a manner driven by increased SCFA, compared to a control group that received two lectures, one on the dietary guidelines and one on exercise [176]. Fecal zonulin and serum lipopolysaccharide-binding protein (markers of intestinal permeability) were decreased by the intervention compared to controls, and this effect was mediated by increased SCFAs [176]. Consistent with many of the effects of Mediterranean dietary interventions, a polyphenol-rich dietary intervention (1391 mg/day total polyphenols) increased the abundance of SCFA-producing bacteria (e.g., *Faecalibacterium*, *Ruminococcaceae*) and reduced serum zonulin levels compared to a control diet consuming half this amount of polyphenols [177]. The improvement in intestinal integrity was highest for those with poorer barrier integrity (higher zonulin levels) at baseline, suggesting a potential floor/ceiling effect. Although both groups were matched for energy, the intervention increased fiber intake making it difficult to determine whether these beneficial effects were due to polyphenols, fiber or their interaction. Mediterranean dietary interventions have been shown to improve depressive symptoms in adults [178]; however, to date, very few published trials report associated changes in the gut microbiota. Some recent dietary interventions report improvements in depressive symptoms after dietary intervention [167,179,180]; one reported increases in probiotic bacteria such as *Bifidobacterium* spp. [179], two report increased microbial diversity [179,180], while another reported no differences in gut microbiota between groups [167]—this is likely due to differences in study design and clinical populations. Indeed, a recent systematic review showed that the Mediterranean diet did not alter the gut microbiota in a consistent manner, despite a number of studies reporting positive changes [181]. The authors highlight heterogeneity across study methodology, intervention design, dietary recommendations, definitions of ‘dietary adherence’ and analysis methods [181]. Another explanation for the lack of a consistent effect may relate to the use of an inconsistent comparison condition, with a variety of dietary types being used as controls across studies, and in some studies, the comparison was relative to baseline.

The low FODMAP diet is a therapeutic approach diet aiming to alleviate GI symptoms by restricting fermentable carbohydrates that have a prebiotic effect and increase gas production, shown to be effective in improving symptoms in irritable bowel syndrome (IBS) [182]. However, due to the low-fiber and low-prebiotic intakes with this diet, bifidobacteria are reduced [76], and SCFA concentrations may be reduced. The addition of a bifidobacteria supplement to a low FODMAP diet may help restore key species [183]. Due to the restrictive nature of elimination diets, long-term use of a low FODMAP diet may compromise nutrition status due to the lower fiber and energy intake [184]. In IBD, low FODMAP dietary interventions do not appear to reduce markers of inflammation, and outcomes for IBD symptom relief were inconsistent across studies, perhaps due to the placebo effect of receiving the sham diet [184]. In an alternate approach, a small RCT showed that a Mediterranean dietary intervention improved IBS symptom scores and depressive scores in IBS patients compared to a control diet matched for FODMAPs [167]. Dietary management of bowel conditions is highly complex and should be managed with clinical oversight and monitoring for patient safety [185].

Although the gut microbiota can respond to dietary change within 24 h [186], it may only be transient if the dietary changes are not sustained [187]. Additionally, it is worth noting that microbial changes in response to diet can be highly individual and vary depending on the habitual/baseline diet [188]. For example, if fiber is lacking from the baseline diet, then the baseline gut microbiota may be restricted in their ability to degrade fiber and produce SCFA and may not respond strongly to a high fiber/plant-based diet [189]. This has led to recommendations to stratify participants in dietary interventions based on the stability of their baseline gut microbiota to enhance predictability of the intervention [187]. Furthermore, some gut microbiota communities may be ‘resilient’ to dietary change—specifically, communities with high functional redundancy and lower diversity [190]. Taken together, these factors highlight the complications involved with dietary intervention approaches to gut microbiota modulation and may explain inconsistencies between studies.

### 5.2. Probiotics, Prebiotics, Paraprobiotics, Postbiotics and Fermented Foods

Probiotics are live microorganisms administered in sufficient amounts to the host for health benefit [191]. Prebiotics are food compounds indigestible by the host that can be acted upon by microbiota and modulate their composition [192,193]. Post-biotics describe metabolic products or byproducts secreted by microbes, or released through cell lysis, such as enzymes, peptides, organic acids, or cell surface proteins [194]. Paraprobiotics are inactivated or heat-killed probiotic organisms, or fragments of them, which can be more safely used by immunocompromised individuals, children, and those with impaired gastrointestinal barrier integrity [193,194]. Synbiotics often refer to a probiotic-prebiotic blend which conveys a synergistic benefit on host health [192,193].

A meta-analysis of studies using probiotics and/or synbiotics and measuring inflammatory markers such as CRP, tumor necrosis factor (TNF)-alpha, and IL-6 showed that probiotic/symbiotic supplementation was able to decrease serum CRP and TNF-alpha in healthy subjects and people with a range of inflammatory conditions, with the greatest effect sizes for both being seen in subjects with IBD [192]. Probiotics, prebiotics, and synbiotics show promise for inducing and maintaining IBD remission and ulcerative colitis symptoms, and particularly synbiotics may increase the relative abundance of some probiotic taxa (e.g., bifidobacteria) in patients with IBD [195]. A meta-analysis has also reported that prebiotics, probiotics and symbiotics reduce depressive symptoms in adults with mild and moderate depression compared to placebo [196], suggesting potential in directly targeting the gut microbiota. However, whilst probiotics show promise for improving various aspects of health, it is unclear whether they actually alter microbiota composition in otherwise healthy people, as a systematic review of eight studies identified that only one study saw microbiota differences between those receiving the placebo and the active probiotic [197]. Similarly, several reviews have stated that there is minimal evidence of any benefit from probiotic supplementation in restoring microbiota composition after disturbances such as antibiotic use [198,199]. The small numbers of studies included in these reviews highlight an evidence gap regarding the gut microbiota-modulatory properties of probiotics in healthy people. Hence, whilst probiotic supplementation may confer benefits to certain body systems or alleviate symptoms of underlying health conditions, the evidence is not currently strong enough to suggest widespread use in healthy people [200].

Prebiotics may be a more pertinent avenue for shifting microbiota composition in otherwise healthy individuals, as being microbiome-modulating is a core component of their definition [192,193]. Prebiotics such as fiber can influence gut microbiota composition, but these effects are modulated by baseline fiber intake [201]. The capacity for utilizing these materials needs to be present within the microbial community for them to deliver their selective effect [201]. However, community metagenomic capacity for carbohydrate utilization has been observed in response to a high-fiber diet intervention when baseline fiber was low, despite no changes in microbial diversity [202]. A potential reason for the mixed responses to ‘biotics observed in studies of healthy volunteers may be a lack of accounting for baseline diet when designing the interventions.

Fermented foods, defined as “foods made through desired microbial growth and enzymatic conversions of food components” [203], have also gleaned research interest recently. Some, but not all, fermented foods contain probiotic organisms and postbiotics [203], and many are enriched in prebiotics and parabiotics [204]. Fermented foods may have the potential to enrich nutrition through increasing the bioavailability of nutrients [203], as well as modulate inflammatory processes through their impact on microbiome composition and function [204]. Fermented foods show promise for directly modulating gut microbiome composition and inflammatory markers as an RCT of a fermented food diet identified shifts in microbiota diversity and composition and decreases in IL-6, IL-10, and IL-12β and other inflammatory factors over the 17-week intervention [202]. However, the utility of fermented foods is limited by the unknown quantities of their ‘biotic’ components, as well as with potential safety concerns such as the presence of biogenic amines—a metabolic byproduct occasionally found in fermented foods and associated with some side effects and reductions in medication effectiveness [204]. Further research is needed to ensure the safety and efficacy and appropriate ‘doses’ of these foods before they can be recommended.

### 5.3. Fecal Microbiota Transplant

Fecal microbiota transplant (FMT) describes the administration of typically healthy donor stool into a recipient’s intestinal tract, often with the aim of normalizing the composition and function of the recipient’s gut microbiome [205,206]. It is highly studied and accepted for use in the treatment of recurrent *Clostridium difficile* infection (R-CDI) but is being investigated as a treatment for other microbiota-related disorders [205,206]. FMT is greatly effective against R-CDI, with recipient microbiota being shown to closely resemble that of the donor as early as 24 h post-procedure, allowing the new microbiota to out-compete *C. difficile* for colonization real estate and enlist immune activity against the pathogen [205]. A metric used to assess the success of FMT is microbial strain-sharing between donor and recipient, otherwise known as engraftment [206]. Multiple factors may increase FMT success, including using multiple routes of administration (e.g., both oral and anal routes), intake of antibiotics prior to FMT to inhibit underlying microbiota, and using a donor related to the recipient (e.g., cohabitating) [206]. These factors are associated with both greater changes in the microbiota and better clinical outcomes [206].

Whether non-communicable disease phenotypes can be transferred via FMT is up for debate. FMT from humans with a condition of interest in animals is often used to infer a causal role between the gut microbiota and the pathogenesis of disease, and almost always shows an impact of FMT [165]. Thus, there is debate on whether the causal role of disruptions in the gut microbiota on health is accurate or exaggerated in the literature [165,207]. The few studies in humans investigating non-*C. difficile* outcomes show relatively strong evidence for efficacy in ulcerative colitis, but no significant effect on IBS, and too few studies on metabolic or hepatic conditions to make any determinations on efficacy [208]. However, new FMT clinical trials are being initiated using larger sample sizes to try to provide further evidence on the potential efficacy of this treatment modality on microbiota and immune-modulated conditions. There are also calls for greater investigations into the long-term safety, efficacy and suitability of FMT interventions, since the potential for deleterious outcomes means that an abundance of caution is warranted [209].

### 5.4. Bacteriophage Therapy

Bacteriophages are viruses that target bacteria, displaying strong specificity for particular bacterial populations. They represent a key part of microbiota ecology with the ability to shape microbiota composition [210]. Therapy with preparations of bacteriophages has a long history, much of it devoted to the control of bacterial infections in humans [211]. However, this approach has also been used to modify the gut microbiota to ameliorate relevant diseases, including IBD [212], with potential applications to allergic disorders also identified [213]. It should also be noted that bacteriophages form an important component of fecal microbiota transplants [214]. In addition, phage lysins, the peptide-glycan-degrading enzymes that determine bacterial specificity, are also being developed for microbiota modulation, since they have several advantages in terms of delivery [215].

### 5.5. Pharmacological/Biological Agents

With the growing understanding of the molecular and cellular pathways responsible for relevant diseases, agents specifically targeting these pathways are being utilized. For autoimmune and allergic diseases, the key focus is immune components, for example, anti-CD3 (teplizumab) targeting T cells in type 1 diabetes [216] or anti-IL-13 (tralokinumab) or JAK inhibitor (ruxolinitib) targeting type 2 immunity in atopic dermatitis (AD) [217,218]. However, more exciting is the use of therapies targeting immune pathways in mental health disorders, recombinant IL-1R antagonists (anakinra) and JAK inhibitors targeting inflammation [219].

## 6. Future Challenges

There remain a number of challenges to fully understanding this important area of research. This is due in part to a range of technical challenges that impact precision, reproducibility and level of detail amongst several important parameters. For example, dietary analyses typically involve self-reporting using simplified, generic food intake instruments, which impacts the reliability and granularity of the data [220]. Given the importance of a baseline diet [188] on the microbial response, the lack of appropriate habitual/baseline dietary assessment in intervention trials targeting the gut microbiota makes it difficult to evaluate the true intervention effect on the gut microbiota [185]. Microbiota analysis is highly variable in terms of methodologies used, which can influence the breadth and depth of coverage as well as relative quantitation [221,222]. This results in significant variation in the microbial composition reported between studies, as has been highlighted, for example, in autoimmune rheumatic disease [223]. Immune cell analysis is also variable with respect to the samples analyzed and the markers used, resulting in a patchwork view of the entire immune system [224].

Increased certainty regarding these parameters is essential for implementing axis-based approaches in clinical settings. Firstly, providing evidence identifying the candidate nutrition, microbiota or immune parameter changes that would be needed for therapeutic benefit. Secondly, informing the type of dietary change, microbiota manipulation or immune targeting that would impact the appropriate parameter in an effective manner. Finally, underpinning a more nuanced methodology that also includes gender, age, genetic and environmental factors to facilitate a patient-centered approach. This should extend beyond the fecal microbiota. While its sample collection is relatively easy, non-invasive and repeatable, it does not represent the microbiota across the entire gut, nor that more closely associated with the mucosal surfaces. In addition, the microbiota of other mucosal surfaces should also be considered in particular disease settings, as has been demonstrated for the skin microbiota in allergic dermatitis (AD) [225], the lung microbiota in asthma [139] and AD [225], the sinonasal cavity microbiota in rhinosinusitis [226], and environmental microbiota for multiple diseases [139], not to mention the interplay between these [139,225]. In addition, microbiota analysis has largely concentrated on the bacteria components, which should sensibly be extended to other microbial agents—including fungi, viruses and protozoa. Collectively this is likely to provide additional insights and potentially new therapeutic strategies.

Key to moving the field forward, particularly its incorporation into day-to-day clinical practice, is standardization across characterization and sampling methodologies. Excitingly, clear frameworks/models/recommendations are emerging on multiple fronts, including the holistic evaluation of the microbiota from a genomic [227] or metabolic [228] perspective, a systems approach to immunology [224], recommendations for study design, controls, definitions and analytical methods in feeding trials [181,229] and pre- and pro- biotic studies [230]. Ongoing involvement of relevant clinicians in the generation, endorsement and on-going validation of such recommendations is essential.

## 7. Conclusions

Knowledge of the microbiota has exploded over recent years, including ever-increasing insights into the human gut microbiota. From this has emerged a clear understanding of its central role in an interconnected nutrition–gut microbiota–immunity axis critical for human health. This in turn underpins a new perspective for understanding the etiology and pathogenesis of several human diseases, notably including autoimmune disorders, allergies and mental health disorders. Importantly, it has opened potential new avenues for therapies targeting this axis, such as dietary interventions, fecal microbiota transplant, phage therapy and pharmacological and biologicals that impact immunity. However, more work is required to standardize approaches in the area and further large, well-designed RCT studies are needed to robustly evaluate the opportunities in targeting the nutrition–gut microbiota-immune axis for disease prevention and treatment.

## Data Availability

No new data were created or analyzed in this study. Data sharing is not applicable to this article.
